# Racial and Ethnic Differences in Diabetes Care and Health Care Use and Costs

**Published:** 2006-06-15

**Authors:** Jung-Ah Lee, Chuan-Fen Liu, Anne E Sales

**Affiliations:** University of Washington, School of Nursing, Department of Biobehavioral Nursing and Health Systems; Health Services Research and Development Center of Excellence, VA Puget Sound Healthcare System, Department of Veterans Affairs, University of Washington, School of Public Health and Community Medicine, Department of Health Services, Seattle, Wash; Health Services Research and Development Center of Excellence, VA Puget Sound Healthcare System, Department of Veterans Affairs, University of Washington, School of Public Health and Community Medicine, Department of Health Services, Seattle, Wash

## Abstract

**Introduction:**

Previous studies have shown racial and ethnic differences in diabetes complication rates and diabetes control. The objective of this study was to examine racial and ethnic differences in diabetes care and health care use and costs for adults with diabetes using a nationally representative sample of the U.S. noninstitutionalized civilian population.

**Methods:**

We performed a cross-sectional analysis of the 2000 Medical Expenditure Panel Survey (MEPS) and its related Diabetes Care Survey. The respondents were adults (aged 18 years and older) with diabetes, including non-Hispanic whites, non-Hispanic African Americans, and Hispanics. Racial and ethnic differences were examined in diabetes process of care and health care use and costs using logistic regression, negative binomial regression, and ordinary least squares regression with log cost.

**Results:**

Most of the outcomes in diabetes care management, treatment, and complications were not significantly different among race groups. After adjusting for socioeconomic and demographic characteristics, Hispanics were more likely to have eye problems than whites (odds ratio, 1.56; 95% confidence interval, 1.03–2.56). African Americans and Hispanics had lower total health care costs, lower ambulatory care costs, and lower prescription drug costs than whites (*P* < .01 for all).

**Conclusion:**

We found differences in ambulatory care and prescription drug fills among white, African American, and Hispanic adults with diabetes. However, most of the diabetes care measures were not significantly different among the three racial and ethnic groups. Understanding the reason outcomes do not differ when health care use and costs differ significantly should be a focus of future studies.

## Introduction

Diabetes is one of the most prevalent diseases in the United States (1), resulting in considerable morbidity and mortality and causing an enormous economic burden (2-4). Reducing the incidence of diabetes and its economic burden is one of the goals of the U.S. federal government in *Healthy People 2010* (5). In 2005, approximately 7% of individuals (20.8 million people) in the United States had diabetes; 14.6 million people were diagnosed and 6.2 million people were undiagnosed (1). The prevalence of diabetes varies greatly by race and ethnicity (1,4,6-8). Approximately 13.3% of non-Hispanic African Americans, 9.5% of Hispanics and Latinos, and 8.7% of non-Hispanic whites (all aged 20 years or older) have diabetes (1). Previous studies have shown that African Americans and Hispanics have higher rates of diabetes complications than whites, including end-stage renal disease, retinopathy, blindness, neuropathy, and lower extremity amputation (9-13). Inadequate access to medical care and disparate quality of diabetes care may contribute to racial or ethnic differences in diabetes-related complications (5,14).

Racial and ethnic differences in health insurance coverage for adults with diabetes have been reported (15). In addition, there is evidence of racial and ethnic differences in diabetes care. Non-Hispanic African American women and Hispanic men treated with insulin and oral agents were disproportionately represented among those with poor glycemic control reported in a study using national survey data (9), possibly resulting in increased medical care costs for individuals with diabetes.

Examining racial and ethnic differences in health care use and costs by using a nationally representative sample provides important information for addressing racial and ethnic disparities in health care and may help improve health care delivery. Racial variation in health care services for diabetes care has been examined among elderly Medicare beneficiaries (16). However, few studies compared racial and ethnic differences in health care costs as well as health care use for adults with diabetes in general populations.

Our study had two aims: 1) to examine racial and ethnic differences in diabetes care including diabetes management, treatment, and complications and 2) to examine racial and ethnic differences in health care use and costs among adults with diabetes. We used the 2000 Medical Expenditure Panel Survey (MEPS), a nationally representative sample of the civilian, noninstitutionalized U.S. population, which provides a unique opportunity for studying racial and ethnic differences in diabetes care and health care use and costs.

## Methods

### Data source

The study used data from the household component (HC) of the 2000 MEPS, a survey sponsored by the Agency for Healthcare Research and Quality (AHRQ) and the National Center for Health Statistics (NCHS). We used the 2000 full-year consolidated data files (HC-050) from the MEPS HC survey, which contained questions on demographic characteristics, health conditions, health status, and use and expenditure of medical services. The overall response to the 2000 MEPS was 65.8% (17).

In addition, the 2000 MEPS consolidation file included disease-specific information from a series of surveys that included diabetes. In the beginning of calendar year 2000, MEPS conducted a new series of interviews as part of AHRQ's focus on quality of health care, asking questions about several specific medical conditions such as diabetes, asthma, high blood pressure, heart disease (including coronary heart disease, angina, and myocardial infarction), stroke, emphysema, and joint pain  (17). HC respondents received A Survey About Your Diabetes Care — The Diabetes Care Survey, a self-administered paper-and-pencil questionnaire, if they responded yes to a question about whether their diabetes had been diagnosed by a health care professional.

The Diabetes Care Survey is a series of questions (18) about diabetes management and includes the number of times respondents reported having a glycated hemoglobin A1c (HbA1c) test, the number of times they reported having their feet checked for sores or irritation in 2000, and the last time they reported having an eye examination. Respondents were also asked to report on diabetes treatment (diet, oral medications, and insulin) and complications (eye problems and kidney problems caused by diabetes). Lower extremity amputation, a serious complication of diabetes, and type and duration of diabetes were not included in the Diabetes Care Survey. The response rate for the Diabetes Care Survey was 91.4% (17).

### Study subjects

Out of 24,791 respondents to the 2000 MEPS, 1021 adults (18 years and older) with diabetes responded to the Diabetes Care Survey. Race in this study was defined using two variables in MEPS indicating race (categorized as American Indian, Aleut Eskimo, Asian and Pacific Islander, black, and white) and ethnicity (categorized as Hispanic, non-Hispanic African American, and other). The race variable in this study was categorized into three groups including non-Hispanic white, non-Hispanic African American, and Hispanic. We excluded American Indians, Aleut Eskimos, and Asian Americans and Pacific Islanders because the number of respondents (37 out of 1021 total adult Diabetes Care Survey respondents, or 3.6%) was too small to analyze. Thus, the final sample size was 984 adults (18 years and older) with diabetes, of whom 540 (54.9%) were white, 210 (21.3%) African American, and 234 (23.8%) Hispanic. Population weights from the full-year sample were used to generate estimates for the national population of adults with diabetes as of the index date December 31, 2000. The population weighted study sample represents 16,808 adults with diabetes in 2000.

### Health care use and costs

Health care use included ambulatory care visits and prescription drug fills. Ambulatory care visits in both office-based settings and hospital outpatient settings were included. Physician and nonphysician visits were included in ambulatory care visits. Nonphysicians included nurse practitioners, physician assistants, chiropractors, podiatrists, physical and occupational therapists, and social workers. Prescription fills were defined as all prescribed medications purchased in 2000.

Health care costs in MEPS were defined as direct payments rather than charges by providers, including out-of-pocket costs and third-party payments (17). Administrative costs (i.e., costs not directly related to patient care), over-the-counter drugs, and payments for alternative care services were not included in MEPS. The three components of health care costs in this study included ambulatory care costs, prescription drug costs, and total health care costs.

Ambulatory care costs included payment for providers in office-based settings and hospital-based settings. The cost of prescription fills includes all amounts paid out-of-pocket and by third-party payers for prescribed medications purchased in 2000. Total health care costs were defined as the sum of payments for care for ambulatory care visits, hospital inpatient stays with zero-night admission, emergency department visits, dental care, home health care, and other care including vision aids, medical supplies and equipment, and prescription medications.

### Diabetes care

Process and outcome measures specific to diabetes care included management, treatment, and diabetes-related complications. Diabetes care management included whether the respondents had received an HbA1c test, had had their feet checked for sores or irritation, and had received an eye examination. Diabetes treatment included diet modification, oral medications, and insulin therapy reported by Diabetes Care Survey respondents. The variables for diabetes complications included eye and kidney problems caused by diabetes. In this study, all measures were binary (yes or no) variables using the original MEPS variables in the Diabetes Care Survey for statistical analysis. Positive answers were coded *1;* for example, having received any HbA1c test was coded as *1 *if a Diabetes Care Survey respondent reported having received one or more than one HbA1c test; we recorded *0* otherwise.

### Statistical analysis

Table 1 shows the definitions of dependent and independent variables. The primary independent variable of interest is race. Covariates included age, sex, education, marital status, living in a standard metropolitan statistical area (MSA), income, health insurance, having a usual source of care (USC) provider, self-rated health status, employment, and comorbidity in multivariate analyses. Having a USC is a commonly used measure of access to care.

The data were analyzed using bivariate and multivariate methods with Stata 7 (Stata Corp, College Station, Tex). Chi-square (Χ^2^) tests were used to compare racial and ethnic differences in individual characteristics. Logistic regression was used to examine differences in diabetes care among the three racial and ethnic groups (white, African American, and Hispanic). For multivariate analyses of utilization measures, which are count variables, we used count data methods (19). Because utilization data are usually not normally distributed and tend to have a long heavy right tail, distributions do not satisfy the assumptions for ordinary least squares regression, which include normality, homoscedasticity, and independence of error terms. We used negative binomial regression models to analyze health care use outcomes (20-23). Incidence rate ratios (IRRs) from negative binomial regression models were used to compare incidence rates of ambulatory care use and prescription fills among three racial and ethnic groups. For example, if the IRR for ambulatory care visits among African Americans is 0.8, the interpretation is that being African American decreases the expected number of visits compared with whites by a factor of 0.8, holding other variables constant. In other words, being African American decreases the expected number of ambulatory care visits by 20% (20).

We performed a natural logarithmic transformation of the cost variables to compensate for the nonnormal distribution and high degree of right skewing. In the regression model using the log transformation for costs, we can interpret the coefficients (β) as percent changes for a change in a dummy variable from zero to one by calculating exponentiation of the β coefficient − 1 (exp [β] − 1) (24,25).

All analyses were adjusted for the complex survey design used in MEPS (17). The 2000 MEPS diabetes weights from the Diabetes Care Survey were used for the diabetes care analyses to yield valid estimates. The MEPS person-level weights were used for health care use and cost analyses to yield valid national estimates for individuals with diabetes (17). The University of Washington Human Subjects Committee determined this study to be exempt from human subjects review.

## Results

### Baseline demographic characteristics

Table 2 presents demographic characteristics for adults with diabetes by racial and ethnic group. Among adults with diabetes, we found statistically significant differences across racial and ethnic groups in age, sex, education, marital status, income, insurance, and comorbidity. The mean age of adults with diabetes was 60 (61 among whites, 59 among African Americans, and 57 among Hispanics, *P* = .006). The proportion of women was highest among African Americans (66%), followed by whites (53%) and Hispanics (51%) (*P* = .04). African Americans had the lowest proportion of married individuals (36%), followed by Hispanics (53%) and whites (62%) (*P* < .001). Whites had the highest proportion of individuals with a high school education or more (77%), followed by African Americans (68%) and Hispanics (51%) (*P* < .001). More than half of African Americans (55%) were low income, compared with 45% of Hispanics and 32% of whites (*P* < .001). Most whites (97%) and African Americans (93%) had health insurance at least some of the time in 2000, but only 80% of Hispanics did (*P* < .001). Whites (72%) were significantly more likely to have private insurance than African Americans (50%) or Hispanics (40%) (*P* < .001), whereas African Americans (24%) and Hispanics (28%) were significantly more likely to be enrolled in Medicaid than whites (8%) (*P* < .001). The proportion of individuals having a USC was similar among the racial and ethnic groups (96% for whites, 94% for African Americans, and 90% for Hispanics, *P* = .12). Seventy-one percent of African Americans were concomitantly diagnosed with high blood pressure, compared with 64% of whites and 49% of Hispanics (*P* = .01). Whites had the highest proportion of concurrent heart disease with diabetes (35%), compared with 23% among African Americans and 14% among Hispanics (*P* < .001).

### Diabetes care

Table 3 presents unadjusted diabetes process-of-care measures by racial and ethnic group. Most of the diabetes care measures, including management, complications, and treatment, were not significantly different among racial and ethnic groups. However, dilated-eye examination rates were significantly different among the groups (95% among whites, 90% among African Americans, and 86% among Hispanics, *P* = .02). The probability of having eye problems caused by diabetes also differed significantly across the three groups (23% among whites, 33% among African Americans, and 33% among Hispanics, *P* = .03). 

Table 4 presents adjusted odds ratios (ORs) for diabetes care by racial and ethnic group. After adjusting for covariates, Hispanics were more likely than whites to have eye problems from diabetes complications (OR, 1.56; 95% CI, 1.03–2.56). We found no significant differences in other measures of diabetes management, complications, and treatment and among racial and ethnic groups after adjustment.

### Health care use and costs

Table 5 summarizes unadjusted health care use and costs. The probability of using ambulatory care was significantly different among groups (*P* = .04), but we found no significant difference in the mean number of ambulatory care visits. Whites were more likely to use ambulatory care (96%), followed by African Americans (92%) and Hispanics (89%). We found no significant difference in the probability of having a prescription fill among the groups. However, whites filled more prescriptions (38.3 fills), followed by African Americans (29.8 fills) and Hispanics (29.8 fills) (*P* = .002).

We found significant differences in ambulatory care costs and prescription drug costs among the three groups. Whites had the highest ambulatory care costs ($1783), followed by African Americans ($1654) and Hispanics ($1028) (*P* = .03). The prescription drug costs among the three groups were $1886 for whites, $1419 for Hispanics, and $1392 for African Americans (*P* < .001). Although we found no significant difference in total health care costs among the racial and ethnic groups, the proportion by level of payment sources to total expenditure was different. Approximately one third of total health care costs was paid by private insurance among whites. Medicaid paid more health care costs for African Americans ($985) and Hispanics ($803) than whites ($374) (*P* = .01).

Table 6 presents adjusted health care use and costs. Compared with whites, African Americans were less likely to have ambulatory care use (IRR, 0.71; 95% CI, 0.58–0.87) and prescription fills (IRR, 0.73; 95% CI, 0.63–0.83). After adjustment, we found no significant differences between whites and Hispanics in the probability of having ambulatory care use and prescription fills.

Compared with whites, African Americans had 25% lower total health care costs (*P* = .008), 51% lower ambulatory care costs (*P* < .001), and 36% lower prescription drug costs (*P* < .001). Hispanics also had 31% lower total health care costs (*P* = .006) and 58% lower ambulatory care costs (*P* = .003) than whites.

## Discussion

Using a nationally representative sample to examine variation across racial and ethnic groups in diabetes process of care, as well as health care use and costs for adults with diabetes, we found that self-reported processes of care for diabetes, including management, complications, and treatment, were not significantly different among whites, African Americans, and Hispanics, except in the rate of dilated-eye examination, which was lower among Hispanics. In addition, African Americans and Hispanics incurred lower health care costs than whites.

Consistent with previous studies (26,27), we did not find significant differences among the three groups in HbA1c testing or examining feet for sores. These results may be related to the characteristics of the study sample participants, most of whom had insurance (94%). Most also had a USC provider (95%). However, this study found a higher proportion of adults (more than 90%) with diabetes reporting having had a dilated-eye examination in 1 year than a study using data from the Third National Health and Nutrition Examination Survey (NHANES III), which found a rate of 60% to 70% (26). Some published studies have found that Hispanics have a higher prevalence of diabetic retinopathy than non-Hispanic whites (28,29), and this study also found that Hispanics were more likely to have self-reported eye problems than whites. However, no significant difference was found in self-reported eye problems between African Americans and whites in this study. This finding is similar to the findings in the Harris et al study, which also used NHANES III data (13). 

A study by Karter et al (10), which was based on patients from Kaiser Permanente Medical Care Program in northern California, found that African Americans and Hispanics had higher rates than whites of microvascular complications, in particular end-stage renal disease, but our study did not find a racial and ethnic difference in self-reported kidney problems. The differences in results regarding kidney problems may be a result of differences in the study samples.

We also found no racial and ethnic difference in diabetes treatment with oral agents, insulin, or both. The percentage of those treated with insulin among the groups (white, 28%; African American, 38%; Hispanic, 24%) was similar to the percentage identified in a previous study (26). However, our study found a high proportion (57%) of adults with diabetes taking oral agents alone for glucose control than the study using NHANES III data (26) (which found less than 50% across racial and ethnic groups).

We found that African Americans were less likely to have ambulatory care and prescription fills than whites. Results show significant differences among groups in the proportion of individuals with a high school education or more. These differences in education may affect awareness of health-care–seeking behaviors for preventive care, which may result in differences in use among different races and ethnicities.

After controlling for all other factors, African Americans and Hispanics had significantly lower total health care costs, ambulatory care costs, and prescription costs than whites. Several reasons may explain the cost differences among groups. First, the differences may stem from differences in health care use. Despite similar access to care, we found that African Americans were less likely to have ambulatory care visits and prescription fills than whites (measured by insurance coverage and having a USC). Second, the cost differences may reflect payment differences among types of health insurance coverage. Although the majority of adults with diabetes were insured at some point in 2000 (94%), we found remarkable differences in having private insurance or being on Medicaid among the  groups. Medicaid payments are commonly lower than payments from private insurance. Because health care costs in MEPS were defined as direct payments to providers rather than charges, the lower health care costs among African Americans and Hispanics may partially reflect lower payments from Medicaid. Finally, the cost difference may also reflect access to different types of services caused by differences in insurance coverage among the racial and ethnic groups. Individuals with private insurance may have greater access to higher-cost procedures than those on Medicaid (30). It is also possible that rates of service use accompanying higher costs among white individuals with diabetes may reflect inefficient practice and possibly effects of supplier-induced demand (31).

This study has several limitations. First, diabetes process-of-care measures were self-reported and may be subject to recall bias. However, health care use and costs were validated by direct contact with medical providers, pharmacies, and health insurance companies identified by household respondents in MEPS (17).

Second, some important factors such as the duration, type, and severity of diabetes, which are critical factors for disease-severity adjustment in comparing differences in diabetes care and health care use and costs, were not included in the survey. However, we used self-rated health status and comorbidity to control for case mix.

Finally, individuals with undiagnosed diabetes were not included in MEPS. Therefore, racial and ethnic disparities for individuals with undiagnosed diabetes, which might be more substantial than for those with diagnosed diabetes, are still unknown. Harris et al (26) reported in 1998 that 6% of the U.S. population 40 to 74 years of age had undiagnosed diabetes and 11% of the U.S. population aged 60 to 74 years had undiagnosed diabetes.

Our study provides insight into racial and ethnic differences in diabetes process of care and health care use and costs. African American and Hispanic adults with diabetes had lower health care use and incurred lower costs than whites, particularly in ambulatory care visits and prescription fills. Future studies should focus on the underlying causes of these racial and ethnic differences in health care use among diabetes to reduce racial and ethnic disparities in diabetes care and should include longitudinal, prospective studies to explore the dynamic effects of changes in health insurance and other socioeconomic factors over time.

## Figures and Tables

**Table 1:** Dependent and Independent Variables for Racial and Ethnic Differences in Diabetes Care and Health Care Use and Costs

**Variables^a^**	**Definition**
**Dependent variables**	
Diabetes care	Binary variable (yes = 1, no = 0)
Management	
Hemoglobin A1c	Hemoglobin A1c test
Feet check	Feet checked for sores
Eye examination	Eye examination with pupils dilated
Complication	
Kidney problems	Kidney problems caused by diabetes
Eye problems	Eye problems caused by diabetes
Treatment	
Diet modification	Diet modification
Oral medication	Oral medication
Insulin	Insulin injection
Health care use	
Ambulatory care visits	Number of visits (physician and nonphysician) to office-based and hospital outpatient settings
Prescription fills	Number of all prescribed medications purchased in 2000
Health care costs	
Total health care costs	Sum of all costs for ambulatory care visits, prescription fills, inpatient care, emergency department visits, dental care, and home health care
Ambulatory care costs	Costs of ambulatory care visits (physician and nonphysician) in office-based and hospital outpatient settings
Prescription drug costs	Costs paid out-of-pocket and by third-party payers for all prescribed medications purchased in 2000
**Independent variables**	
Race	
White	1 = non-Hispanic white; 0 = otherwise
Black	1 = non-Hispanic African American; 0 = otherwise
Hispanic	1 = Hispanic; 0 = otherwise
Age	18 years or older
Elderly	1 = 65 years or older; 0 = otherwise
Sex	1 = female; 0 = male
Education	1 = high school or more;0 = less than high school
Marital status	1 = married; 0 = widowed, divorced, separated, or never married
Metropolitan statistical area (MSA)	1 = living in MSA; 0 = otherwise
Income	1 = ≥200% of poverty line; 0 = <200% of poverty line
Employment	0 = unemployed during all of 2000; 1 = otherwise
Health insurance status	1 = having health insurance any time in 2000; 0 = otherwise
Usual source of care (USC)	1 = having a USC provider; 0 = no
Perceived health status (physical and mental health status)	1 = self-ranked excellent, very good, or good;0 = fair or poor
Comorbidity	1 = having any chronic conditions including high blood pressure, heart diseases (e.g., angina, myocardial infarction, coronary, other), or stroke; 0 = none of above

a Covariates in multivariate regression analyses.

**Table 2:** Baseline Demographic Characteristics of Adults With Diabetes, Based on Data From Household Component of 2000 Medical Expenditure Panel Survey^a^

**Characteristic**	**White (n = 540)**	**African American (n = 210)**	**Hispanic (n = 234)**	**Total (N = 984)**	***P* Value**
Age, y, mean (SE)	60.7 (0.81)	58.7 (0.94)	56.8 (1.17)	59.9 (0.65)	.006
Elderly (65 y or older), %	45	37	37	43	.22
Female, %	53	66	51	55	.04
High school education or above, %	77	68	51	72	<.001
Married, %	62	36	53	57	<.001
Living in metropolitan statistical area, %	72	80	90	75	.08
Income					
Middle/high, %	68	45	55	62	<.001
Low/poor, %	32	55	45	38	
Unemployed in all of 2000, %	55	58	53	55	.84
Insured any time in 2000, %	97	93	80	94	<.001
Payment source					
Medicare, %	52	46	42	50	.10
Medicaid, %	8	24	28	13	<.001
Private insurance, %	72	50	40	64	<.001
With a usual source of care provider, %	96	94	90	95	.12
Good/excellent perceived physical health, %	62	58	55	61	.64
Good/excellent perceived mental health, %	87	78	84	85	.09
Comorbidity					
High blood pressure, %	64	71	49	64	.01
Heart disease (angina, myocardial infarction, coronary, other), %	35	23	14	31	<.001
Stroke, %	8.8	8.4	8.1	8.6	.99
Any comorbidity conditions above, %	75	74	53	72	<.001

a The results are adjusted by population weights.

**Table 3:** Racial and Ethnic Differences in Diabetes Process of Care Among Adults With Diabetes, Based on Data From Household Component of 2000 Medical Expenditure Panel Survey^a^ (N = 984)

**Care Category**	**White, %**	**African American, %**	**Hispanic, %**	**Total, %**	***P* Value**
**Management**					
Hemoglobin A1c	91	86	85	89	.26
Feet checked	68	60	66	66	.48
Dilated-eye examination	95	90	86	93	.02
**Complications**					
Kidney problems	15	14	19	15	.81
Eye problems	23	33	33	26	.03
**Treatment**					
Diet modification	83	82	83	83	.99
Oral medication	70	74	76	72	.65
Insulin	28	38	24	29	.09

a The results are adjusted by population weights.

**Table 4:** Racial and Ethnic Differences in Diabetes Process of Care Among Adults With Diabetes, Adjusted,^a^ Based on Data From Household Component of 2000 Medical Expenditure Panel Survey

**Care Category**	**White OR (95% CI)**	**African American OR (95% CI)**	**Hispanic OR (95% CI)**
**Management**			
Hemoglobin A1c	Ref	0.62 (0.31-1.23)	0.98 (0.42-2.25)
Feet checked for sores	Ref	0.70 (0.45-1.11)	1.04 (0.62-1.74)
Dilated eye examination	Ref	0.54 (0.23-1.29)	0.54 (0.28-1.04)
**Complication**			
Kidney problems	Ref	0.62 (0.32-1.20)	1.04 (0.53-2.08)
Eye problems	Ref	1.33 (0.86-2.03)	1.56 (1.03-2.56)
**Treatment**			
Diet modification	Ref	1.03 (0.67-1.58)	1.60 (0.89-2.88)
Oral medication	Ref	1.27 (0.85-1.87)	1.48 (0.83-2.65)
Insulin	Ref	1.38 (0.89-2.16)	0.69 (0.43-1.12)

OR indicates odds ratio; CI, confidence interval; Ref, reference group.

a Adjusted for age, sex, education, marital status, living in metropolitan statistical area, income status, insurance status, having a usual source of care provider, self-rated health status, and comorbidity. All analyses were also adjusted for population weights.

**Table 5:** Racial and Ethnic Differences in Health Care Use and Costs Among Adults with Diabetes, Based on Data From Household Component of 2000 Medical Expenditure Panel Survey^a^ (N = 984)

**Categories**	**White**	**African American**	**Hispanic**	**Total**	***P* Value**
Any ambulatory care use, %	96	92	89	95	.04
Mean no. of ambulatory care visits	13.6	10.6	10.4	12.6	.06
Any prescription drug use, %	98	98	93	97	.06
Mean no. of prescription fills	38.3	29.8	29.8	35.9	.002
Ambulatory care cost, $ (SE)	1783 (152)	1654 (298)	1028 (230)	1675 (125)	.03
Prescription drug cost, $ (SE)	1886 (99)	1392 (100)	1419 (121)	1748 (74)	<.001
Total cost, $ (SE)	6887 (465)	6162 (860)	5647 (725)	6616 (369)	.35
Average payment by Medicare, $ (%)^b^	2674 (38.8)	1774 (28.8)	2311 (40.9)	2482 (37.5)	.20
Average payment by Medicaid, $ (%)	374 (5.4)	985 (16.0)	803 (14.2)	527 (8.0)	.01
Average payment by private insurance, $ (%)	1981 (28.8)	1184 (19.2)	967 (17.1)	1726 (26.1)	.003
Average payment out of pocket, $ (%)	1371 (19.9)	880 (14.3)	1045 (18.5)	1251 (18.9)	.004

a The results are adjusted by population weights.

b Proportion of total cost.

**Table 6:** Racial and Ethnic Differences in Health Care Use and Health Care Costs Among Adults With Diabetes, Adjusted,^a^ Based on Data From Household Component of 2000 Medical Expenditure Survey

**Categories**	**White**	**African American**	**Hispanic**
Ambulatory care use, IRR (95% CI)	Ref	0.71 (0.58-0.87)	0.82 (0.57-1.18)
Prescription fills, IRR (95% CI)	Ref	0.73 (0.63-0.83)	0.88 (0.75-1.03)
Total health care costs, % change^b^	Ref	–25 (*P* = .008)	–31 (*P* = .006)
Ambulatory care costs, % change^b^	Ref	–51 (*P* < .001)	–58 (*P* = .003)
Prescription drug costs, % change^c^	Ref	–36 (*P* < .001)	–20 (*P* = .24)

Ref indicates reference group; IRR, incidence rate ratio.

a Adjusted for age, sex, education, marital status, living in metropolitan statistical area, income status, insurance status, having a usual source of care provider, self-rated health status, and comorbidity. All analyses were also adjusted for population weights.

b % change between African Americans and whites: exp (β coefficient) − 1.

c % change between Hispanics and whites: exp (β coefficient) − 1.
